# Interleukin 20 receptor subunit beta *(IL20RB)* predicts poor prognosis and regulates immune cell infiltration in clear cell renal cell carcinoma

**DOI:** 10.1186/s12863-022-01076-4

**Published:** 2022-07-26

**Authors:** Haoxun Zhang, Yiwen Liu, Bowen Wang, Chunyang Wang

**Affiliations:** The First Affiliated Hospital of Harbin Medical University, Harbin Medical University, Harbin, Heilongjiang China

**Keywords:** Immune cell infiltration, *IL20RB*, Prognosis, Proliferation, Biomarker

## Abstract

**Background and objective:**

Emerging evidence has proven the robust role of tumor mutation burden (TMB) and immune cell infiltration (ICI) in cancer immunotherapy. However, the precise effect of TMB and ICI on clear cell renal cell carcinoma (ccRCC) remains elusive and merits further investigation. Therefore, we aim to identify the TMB-related genes in predicting prognosis and to explore the potential mechanisms of the identified Interleukin 20 receptor subunit beta (*IL20RB*) in ICI in ccRCC.

**Method:**

The relative information of patients with ccRCC was obtained from The Cancer Genome Atlas database (TCGA). Immune-related genes were downloaded from the Immunology Database and Analysis Portal database. Cox regression analysis was used to identify prognosis-related immune genes for ccRCC. The relationship of *IL20RB* expression levels with clinicopathological parameters was analyzed using the “limma” and “survival” packages. Gene Expression Omnibus (GEO) and International Cancer Genome Consortium (ICGC) databases were used as external validation. Quantitative Real-time PCR (qRT-PCR) and western blots were used to validate the expression levels of *IL20RB* in tumor cells. Cell counting kit-8 (CCK-8) assay and colony formation assay were used to examine the effect of *IL20RB* on the viability of ccRCC cells. Gene set enrichment analysis (GSEA) was introduced for the analysis of *IL20RB*-related signaling pathways. Tumor Immune Estimation Resource (TIMER) and Tumor and Immune System Interaction Database (TISIDB) were utilized to determine the correlation of *IL20RB* expression levels with tumor-infiltrating immune cells (TIICs).

**Results:**

*IL20RB* was significantly overexpressed in different ccRCC tissues and cells. High *IL20RB* expression in ccRCC patients was associated with short overall survival, high tumor grade, and advanced TNM stage. After knockdown of *IL20RB* with small interfering RNA (siRNA) technology, ccRCC cells’ proliferation was significantly attenuated. Moreover, overexpression of *IL20RB* could increase the infiltration level of several immune cells, especially T follicular helper cells (Tfh), and overexpressed Tfh cells were correlated with poor prognosis in ccRCC.

**Conclusions:**

*IL20RB* may function as an immune-associated therapeutic target for it determines cancer progression and regulates immune cell infiltration in ccRCC.

**Supplementary Information:**

The online version contains supplementary material available at 10.1186/s12863-022-01076-4.

## Introduction

Renal cell carcinoma (RCC) ranks among the top ten most frequently diagnosed cancers worldwide, and it accounts for approximately 3% of cancers in adulthood [1, 2]. Clear cell RCC (ccRCC) is the major histopathological subtype of RCC, accounting for nearly 75% of all RCC cases [3]. The main treatments for localized RCC include partial or radical nephrectomy, radiofrequency ablation, and active surveillance (monitoring of tumor growth with periodic radiographic studies) [4–6]. However, the treatment options for advanced ccRCC patients are still very limited, and the 5-year survival rate is only approximately 12% [1, 7].

Recently, immunotherapy has been considered an effective therapeutic method [8], and nivolumab plus cabozantinib was approved in January 2021 by the United States Food and Drug Administration as the first-line therapy for advanced RCC [9]. However, only a limited number of patients benefit from such therapy, while the majority of them fail to respond to treatment [10]. Therefore, it is imperative to explore the molecular mechanism and biomarkers predicting the response to immunotherapy. At present, a series of important molecular determinants, including cytotoxic T lymphocyte antigen-4 (CTLA4), programmed death-ligand 1 (PD-L1), DNA mismatch-repair deficiency, and tumor-infiltrating lymphocytes (TILs), have been identified for this purpose in diverse types of cancer [11–13].

Tumor mutation burden (TMB) refers to the quantity of somatic coding mutations per MB (million bases) [14]. To date, TMB has been implicated in tumorigenesis and predicting the response and survival prognosis to immune checkpoint blockade (ICB) in various types of cancers [15, 16]. A previous study examined the prognostic value of TMB and its potential relationship with immune cell infiltration (ICI) and immunotherapy responsiveness in ovarian cancer [17]. However, whether TMB is associated with prognosis and ICI in ccRCC remains mysterious. Thus, in this research, we took advantage of bioinformatics resources and methods combined with molecular biology to identify and verify that *IL20RB* was an effective prognostic predictor involved in TMB and ICI in ccRCC.

## Materials & methods

### Data acquisition and processing

Gene expression profiles and corresponding clinical data for 539 ccRCC and 72 paracancerous samples were downloaded using the Cancer Genome Atlas (TCGA, http://cancergenome.nih.gov/) database. The format of the downloaded clinical data was “BCR-XML”, and to increase the accuracy of the data, we excluded samples whose follow-up time was < 30 days. Three gene expression profile datasets, GSE40435, GSE46699, and GSE53757, were downloaded from the GEO database (https://www.ncbi.nlm.nih.gov/geo/). The GSE46699 and GSE53757 were based on the GPL570 platform, and the GSE40435 was based on the GPL15008 platform. Additionally, gene expression data and survival information of ccRCC patients were downloaded from the ICGC database (http://icgc.org/). Data were downloaded only from public databases without any ethical conflicts.

### TMB calculation

Somatic mutation data were (*n* = 336) downloaded from TCGA database and the workflow type of was set as “VarScan2 Variant Aggregation and Masking”. Subsequently, we analyzed and visualized the somatic mutation data via the “maftools” package in the R 4.0.3 programming language. According to the median value of TMB, which was acquired based on a calculation of the number of TMBs per MB, the patients were categorized into low-TMB and high-TMB groups. Kaplan–Meier analysis was used to show the survival difference between the high and low TMB expression groups.

### Kyoto Encyclopedia of Genes and Genomes (KEGG) pathway enrichment and Gene Ontology (GO) analyses

The DEGs in the two groups were identified using the “limma” package in the R programming language, and the thresholds were set to *P* < 0.05 and |Log FC | > 1. KEGG pathway enrichment and GO analyses were conducted using the R programming language to investigate the potential roles of DEGs [18–20].

### Cox regression analysis

Immune-related genes were downloaded from the Immunology Database and Analysis Portal (ImmPort, http://www.immport.org/) database. Venn diagrams exhibited the immune-related DEGs. Cox regression analysis was used to identify prognosis-related immune genes for ccRCC, and forest plots were drawn with the Sangerbox online tool (http://www.sangerbox.com/tool).

### Identification and validation of prognosis-related immune genes

Gene Expression Profiling Interactive Analysis (GEPIA, http://gepia.cancer-pku.cn/index.html) was utilized to analyze gene expression levels and plot survival curves. The University of ALabama at Birmingham CANcer data analysis Portal (UALCAN, http://ualcan.path.uab.edu/home) was used to further compare the levels of expression and promoter methylation of *IL20RB* between normal and tumor tissues. Survival analysis was performed to determine whether there was a difference in survival rates between different *IL20RB* expression-dependent groups. The “limma” and “survival” packages in the R programming language were used to analyze the relationship of *IL20RB* expression levels with clinicopathological parameters. GEO and ICGC databases were used to validate the expression and survival difference of *IL20RB* in ccRCC.

### Cell cultures

Human ccRCC cell lines 786–0 and normal control cells, Human kidney 2 (HK-2) cells, were obtained from the Cell Resources Center of the Chinese Academy of Sciences (Shanghai, China). A498 and RC-2 cancer cells were obtained from Procell Life Science&Technology Co., Ltd. (Wuhan, China). All cancer cells were cultured in MEM with a 10% serum concentration, 100 U/mL penicillin, and 0.1 mg/mL streptomycin (Gibco, Invitrogen, Carlsbad, CA, USA). HK-2 cells were cultured in RPMI-1640 with a 10% serum concentration, 100 U/mL penicillin, and 0.1 mg/mL streptomycin (Gibco, Invitrogen). Cells were incubated in a humidified incubator at 37 °C with 5% CO2.

### Cell transfection

Lipofectamine 2000 transfection kits were used for transfection. We performed qRT-PCR to evaluate the transfection efficiency after transfecting for 48 h. The siRNA sequences were synthesized by: for si-*IL20RB*#1, 5′-CUGGAGAAACAGUGUACUATT-3′, forward, 5′-UAGUACACUGUUUCUCCAGTT-3′, reverse; for si-*IL20RB*#2, 5′-CUAGAAGAAAUCUGGACAATT-3′, forward, 5′-UUGUCCAGAUUUCUUCUAGTT-3′, reverse; for Si-NC, 5′-UUCUCCGAACGUGUCACGUTT-3′, forward, 5′-ACGUGACACGUUCGGAGAATT-3′, reverse.

### RNA extraction and qRT-PCR analysis

Total RNA was extracted from cells that were washed with cold PBS solution twice using TRIzol RNA extraction reagent according to the manufacturer’s instruction. The cDNA was reversely transcribed using a reverse transcription kit. SYBR Green qPCR was used to evaluate the expression levels of *IL20RB.* The expression of GAPDH was used as the internal control. Primer sequences were as follows: the *IL20RB* primers, forward: 5′-AGGCCCAGACATTCGTGAAG-3′, reverse: 5′-CGACCACAAGGATCAGCATGA-3′; and GAPDH primers, forward, 5′-GGAGCGAGATCCCTCCAAAAT-3′, reverse: 5′-GGCTGTTGTCATACTTCTCATGG-3′. The qRT-PCR system was QuantStudio 3, and the data were analyzed using the 2-ΔΔCT method.

### Western blot

Total protein lysates were isolated from cell lines by treating with the RIPA lysis buffer supplemented with phenylmethanesulfonyl fluoride and phosphatase inhibitor and centrifuged at 12000 rpm at 4 °C. After being separated by 10% SDS-PAGE, the protein samples were transferred onto the PVDF membrane by the wet transfer method. After incubation with 5% skimmed milk for 1 hour at room temperature, membranes were incubated with diluted rabbit primary antibodies: *IL20RB* antibody (A7980, ABclonal), and GAPDH antibody (A19056, 1:1000). Then, the membranes were washed with PBS and incubated with secondary antibody horseradish peroxidase-conjugated goat anti-rabbit immunoglobulin G (Transgene Biotech) for 1 hour. Enhanced chemiluminescence fluorescent detection kit (BB-3501, Amersham Pharmacia) was used to visualize the immunocomplexes and image analysis system (Bio-Rad Laboratories), and Quantity One version 4.6.2 software (Bio-Rad Laboratories) was used to quantify the band intensities.

### CCK-8 assay

The cells were placed in 96-wells plates and treated for 24 h after transfection with siRNA. Then, the CCK-8 reagent was added into cells for another 2 h culture. And the optical density (OD) value was examined with a microplate reader at 450 nm.

### Clone formation assay

The cells at logarithmic phase were suspended and added in a six-well plate at a density of 1 × 10^3^/well, which were incubated at 37 °C for 10 days. When macroscopic clones appeared in the plate, the culture was terminated. The clones were washed with PBS twice and fixed with 4% paraformaldehyde (Sangon Biotech, Shanghai, China) for 15 min and stained with Giemsa stain (Solarbio, Beijing, China) for 10 min.

### Gene Set Enrichment Analysis (GSEA)

GSEA was performed to analyze the *IL20RB*-related signaling pathways with GSEA 4.1.0 software. “c2.cp.kegg.v7.4.symbols.gmt” was selected as the reference gene.

### Correlation between *IL20RB* expression levels and tumor-infiltrating immune cells (TIICs)

TIMER (http://timer.cistrome.org) and TISIDB (http://cis.hku.hk/TISIDB/) were utilized to determine the correlation of *IL20RB* expression levels with TIICs. Additionally, the association between TIICs and prognosis and the correlation between *IL20RB* and immune cell markers were investigated by the ‘Outcome module’ and ‘Gene_Corr module’ of the TIMER database.

### Statistical analysis

The experimental data were analyzed with GraphPad version 8 and R programming language. T-test and *Wilcoxon rank-sum* test were used to compare the difference between 2 groups, and the difference between 2 or several groups was compared with the *Kruskal-Wallis* test. *P* < 0.05 was considered to indicate a significant difference.

## Results

### Landscape of somatic mutations in ccRCC

**Fig. 1:**
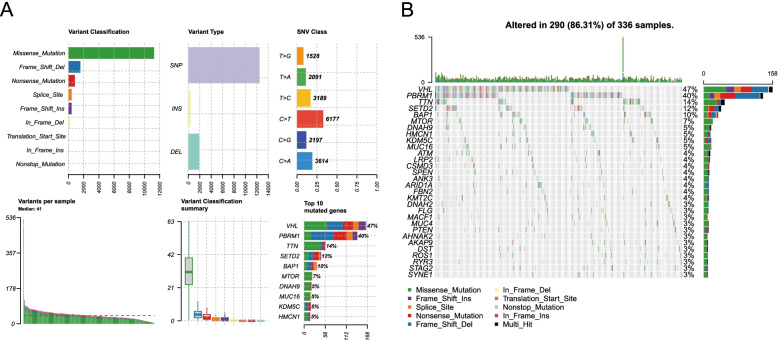
Comprehensive profiling for somatic mutation data. **A** Upper part (from the left to the right) displayed the variant class, variant type, and SNV class. Bottom part (from the left to the right) showed TMB in specific cases and top ten mutated genes in ccRCC. **B** Waterfall plot exhibited the top ten mutant genes in ccRCC, and various colors represented different types of mutation

A total of 339 somatic mutation data points from TCGA were downloaded and analyzed by the R language “maftools” package. The missense mutation accounted for the highest proportion among all variants, and single-nucleotide polymorphisms (SNPs) occurred more frequently than insertions (INSs) and deletions (DELs). In addition, it was revealed that the most frequent SNV (single-nucleotide variant) in ccRCC was C > T, and the number of mutations in each case was displayed, with a median value of 254 (Fig. 1A). In ccRCC samples, the 5 genes with the highest mutation rates were *VHL* (47%), *PBRM1* (40%), *TTN* (14%), *SETD2* (12%) and *BAP1* (10%) (Fig. 1B).

### Correlation analysis of TMB with clinicopathological parameters

**Fig. 2:**
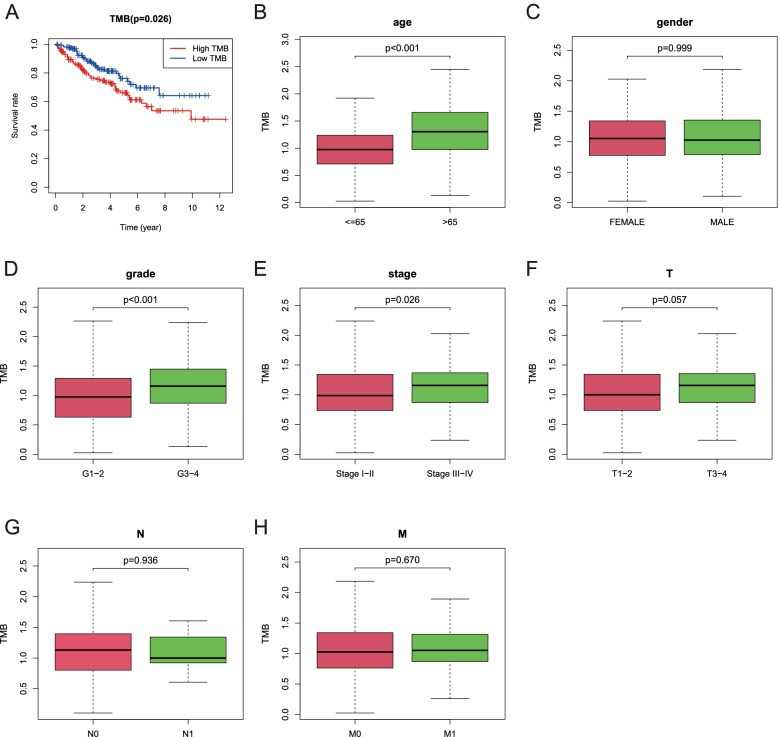
TMB value was associated with clinical characteristics. **A** The survival curves for high-TMB and low-TMB groups. **B, D, E** A high TMB value was correlated with age, tumor grade, and AJCC-stage. **C, F, G, H** TMB value was not associated with gender and TNM-stage

Transcriptome profiles of 72 healthy controls and 539 ccRCC patients were downloaded from the TCGA database. Moreover, the corresponding clinical data of ccRCC patients (*n* = 537) were obtained. After exclusion of cases whose follow-up time was < 30 days, Table 1 summarized the clinical characteristics of 520 ccRCC patients. According to the median TMB value (1.053 per MB), we divided a total of 336 samples into low-TB (*n* = 175) and high-TMB (*n* = 161) groups. Kaplan–Meier analysis was performed (Fig. 2A), and it was revealed that the 5-year survival rate in the low-TMB group (0.762) was significantly higher than that in the high-TMB group (0.661, *p* = 0.026), implying that patients who had low TMB values possessed a better prognosis. In addition, among the 7 clinical characteristics, age (*p* < 0.001), tumor grade (*p* < 0.001) and AJCC-stage (*p* = 0.026) were also correlated with the TMB value (Fig. 2B, D, E). Nevertheless, we did not find a significant difference between the TMB value and other clinicopathological parameters (Fig. 2C, F, G, H). Thus, TMB was deemed a prognostic factor for ccRCC.

**Table 1:** Clinical characteristics of 520 ccRCC cases downloaded from TCGA database

Variable	Proportion of patients (%)
**Age, years old**	
< =65	344 (66.2)
> 65	176 (33.8)
**Gender**	
Male	339 (65.2)
Female	181 (34.8)
**Grade**	
G1	12 (2.3)
G2	222 (42.7)
G3	202 (38.9)
G4	76 (14.6)
Unknown	8 (1.5)
**Stage**	
I	259 (49.8)
II	56 (10.8)
III	119 (22.9)
IV	83 (16.0)
Unknown	3 (0.5)
**T Stage**	
T1	265 (51.0)
T2	68 (13.1)
T3	176 (33.8)
T4	11 (2.1)
**N Stage**	
N0	230 (44.2)
N1	17 (3.3)
Unknown	273 (52.5)
**M Stage**	
M0	413 (79.4)
M1	79 (15.2)
Unknown	28 (5.4)

### DEGs, GO, and KEGG pathway enrichment analyses

**Fig. 3:**
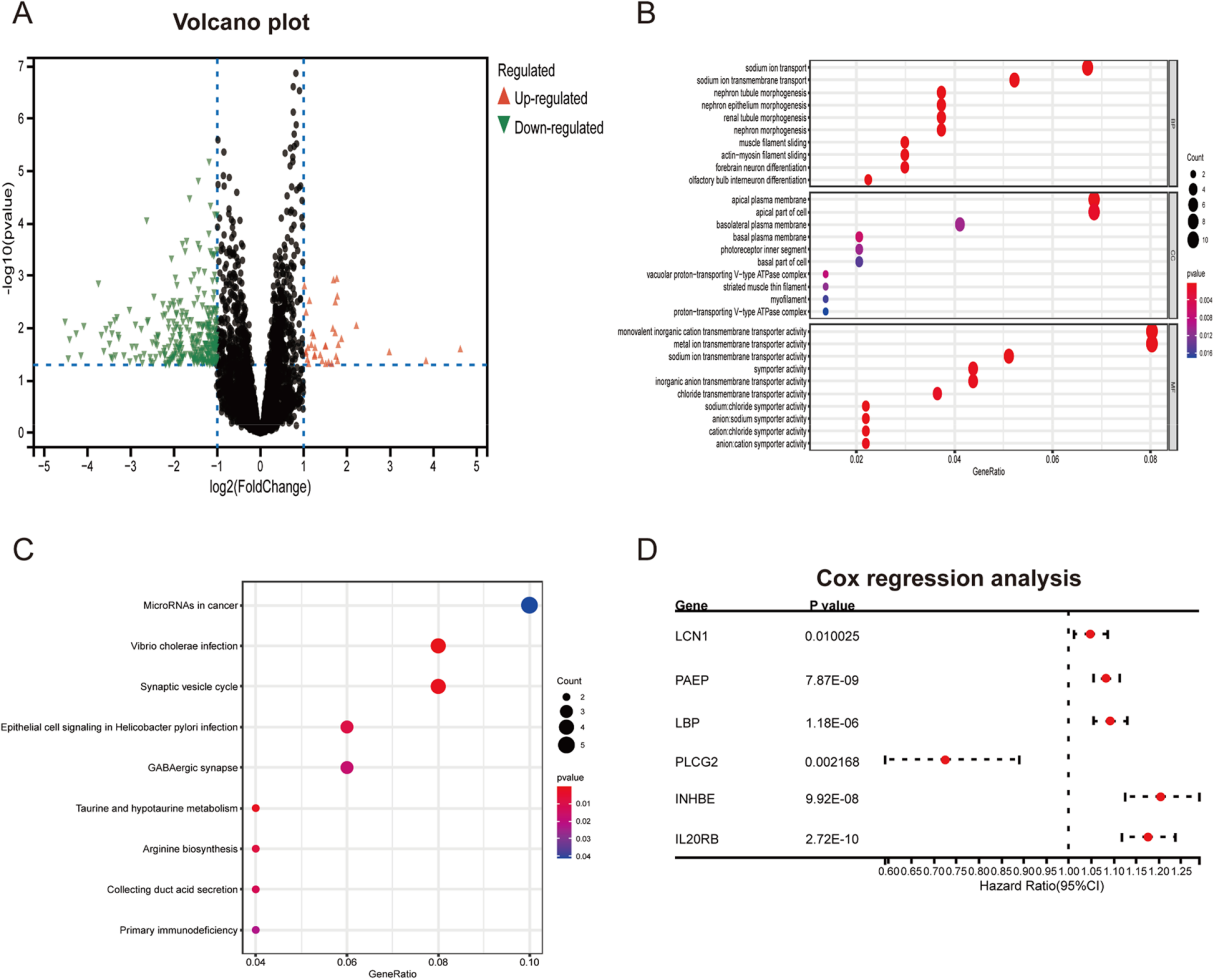
Transcriptome analysis of two TMB-based groups. **A** Volcanic maps for DEGs. Red dots, upregulated genes. Green dots, downregulated genes. Black dots, nondifferentially expressed genes. **B** GO functional analysis. **C** KEGG pathways enrichment analysis. **D** Forest plot illustrating prognosis-related immune genes

We performed differential expression analysis to identify DEGs in the two groups. A total of 340 DEGs were detected (|Log FC| > 1, *p* < 0.05), including 35 upregulated and 305 downregulated DEGs, and the Volcano plot of DEGs was shown in Fig. 3A. According to the results of GO functional analysis, sodium ion transport, chloride symporter activity, and apical plasma membrane were enriched (Fig. 3B). Based on the KEGG pathway enrichment analysis, *Vibrio cholerae* infection, synaptic vesicle cycle, and primary immunodeficiency were the top enriched pathways (Fig. 3C). To explore immune-related DEGs, we downloaded immune-related genes from the ImmPort database. The Venn diagram showed 13 genes that were common between the DEGs and immune-related genes (Fig. S1). Then, prognosis-related immune genes were identified. Finally, 6 prognosis-related immune genes, including *LCN1*, *PAEP*, *LBP*, *PLCG2*, *INHBE*, and *IL20RB*, were identified (Fig. 3D, Table 2).

**Table 2:** Results of the univariate Cox regression analysis

Gene	HR.95 L	HR	HR.95H	***P***-value
**LCN1**	1.011	1.048	1.085	*
**PAEP**	1.054	1.082	1.112	***
**LBP**	1.054	1.092	1.131	***
**PLCG2**	0.591	0.725	0.891	**
**INHBE**	1.124	1.204	1.289	***
**IL20RB**	1.118	1.176	1.236	***

^*^P < 0.05

^**^P < 0.01

^***^P < 0.001

### The *IL20RB* level was strongly correlated with the clinicopathological features of ccRCC

**Fig. 4:**
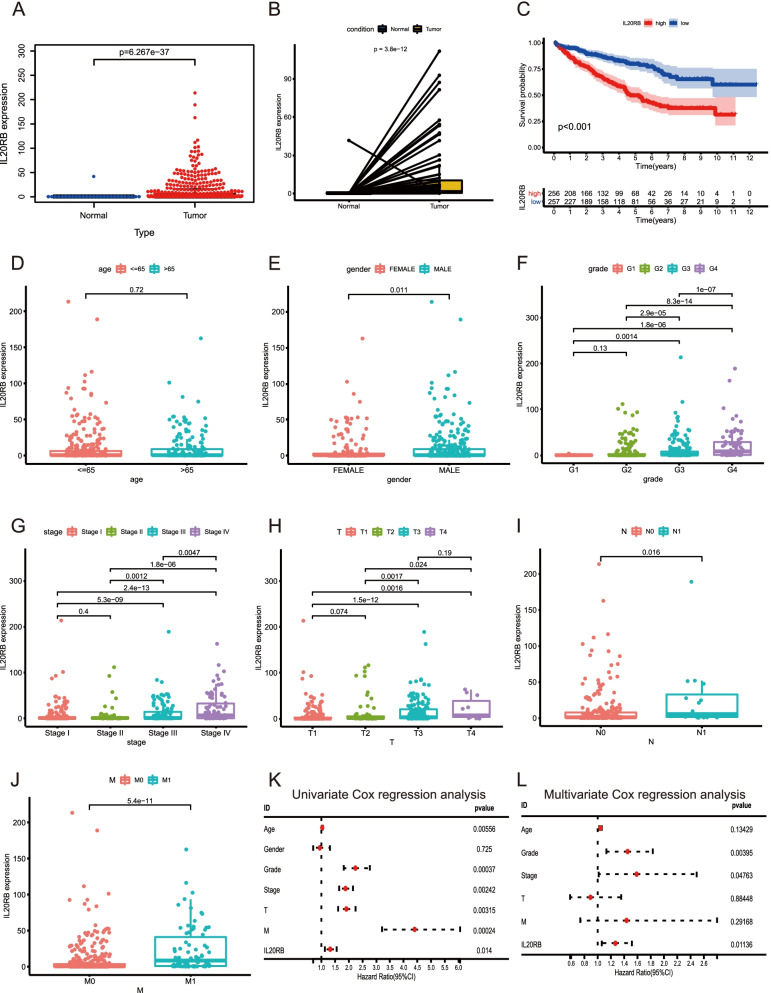
The overexpressed *IL20RB* was associated with clinicopathological parameters. **A** Differential expression analysis of *IL20RB* in ccRCC and normal samples. **B** Pairwise boxplot **(C)** Relationship of *IL20RB* expression levels with survival of ccRCC cases. **D-J** Correlation analysis between *IL20RB* expression levels and clinicopathological parameters. **K, L** The Cox regression analysis of clinicopathological parameters and *IL20RB* expression levels

To further evaluate the prognostic potential of DEGs, we utilized the GEPIA online database to analyze the gene expression levels and to plot survival curves (Fig. S2). Only *IL20RB* exhibited a satisfactory result. Differential expression analysis revealed that the *IL20RB* expression level was notably higher in tumor samples than in normal samples (Fig. 4A, B). The survival curves demonstrated that cases with overexpressed *IL20RB* had shorter overall survival (OS) than those with lower expression (*p* < 0.001, Fig. 4C). Furthermore, we investigated whether *IL20RB* expression was related to the clinicopathological features of ccRCC and found that *IL20RB* overexpression was associated with male sex (*p* = 0.011, Fig. 4E), tumor grade (*p* < 0.001, Fig. 4F), AJCC-stage (*p* < 0.001, Fig. 4G), T stage (*p* < 0.05, Fig. 4H), N stage (*p* < 0.05, Fig. 4I), and M stage (*p* < 0.001, Fig. 4J). However, we found no significant association between *IL20RB* expression and age (*p* = 0.72, Fig. 4D). Cox regression analysis was additionally conducted to indicate whether the *IL20RB* expression level was an independent prognostic factor of cases with ccRCC. As shown in Fig. 4K and L, the *IL20RB* expression level was significantly associated with OS in ccRCC. Collectively, the *IL20RB* expression level was an independent prognostic factor of ccRCC.

### External validation of *IL20RB* in ccRCC

**Fig. 5:**
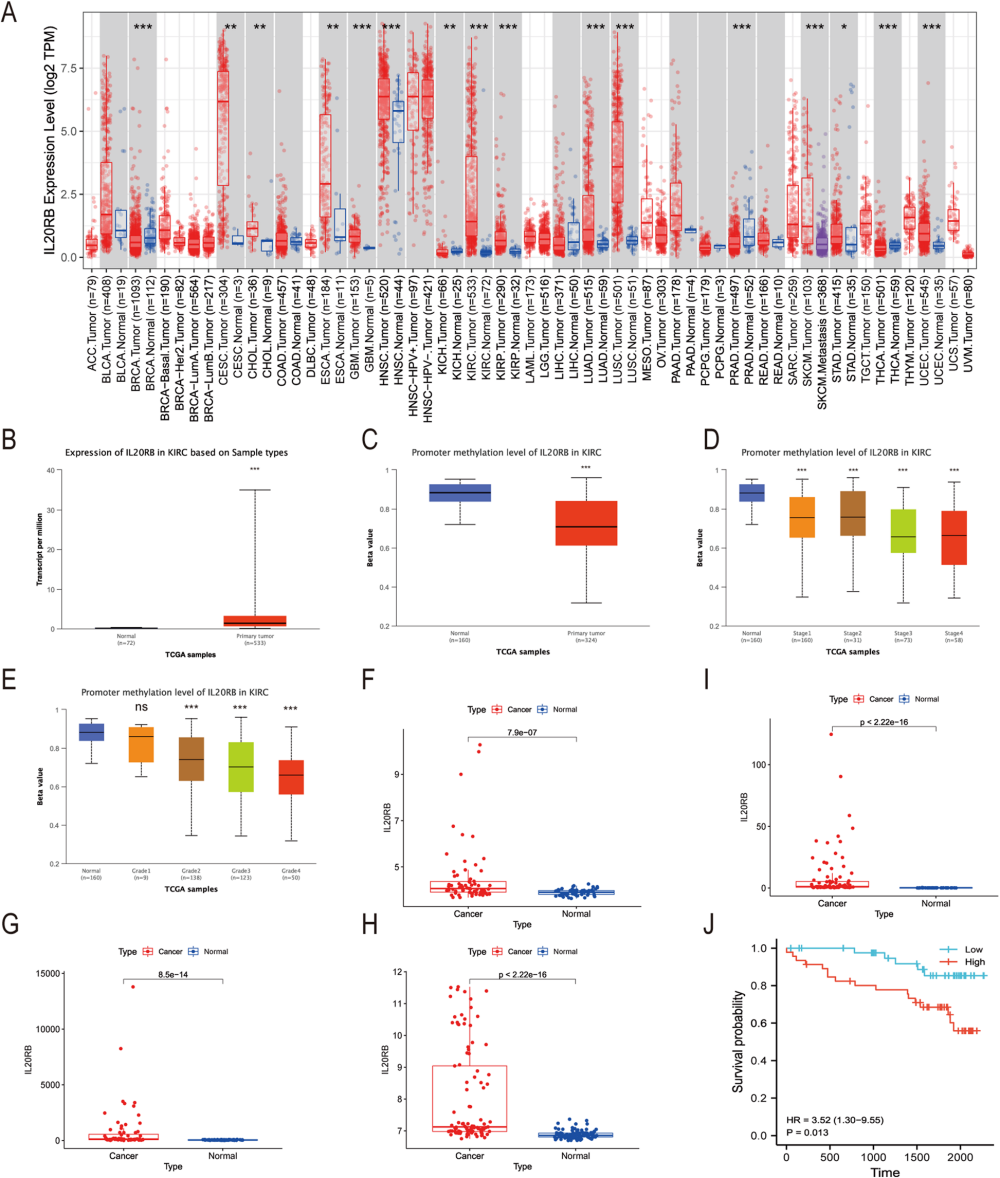
Differential expression and survival analysis validation of *IL20RB* in ccRCC **(A)** Expression level of *IL20RB* in Pan-cancer perspective analyzed through TIMER database. **B-E** Expression and promoter methylation levels of *IL20RB* in ccRCC analyzed through UALCAN database. **F-H** Differential expression of *IL20RB* in GEO (GSE40435, GSE46699, and GSE53757). **I-J** Expression and survival analysis of *IL20RB* in ICGC. *, *P* < 0.05; **, *P* < 0.01; ***, *P* < 0.001; **** *P* < 0.0001

Then, we used the ‘Gene DE module’ of the TIMER database to analyze the differential expression of *IL20RB* in pan-cancer. As shown in Fig. 5A, the expression levels of *IL20RB* were significantly increased in multiple cancer types, including kidney renal clear cell carcinoma (KIRC) (*p* < 0.001). The online database UALCAN further validated that the expression and methylation levels of *IL20RB* were different between kidney normal and tumor tissues. The results showed that *IL20RB* was overexpressed in tumor tissues and promoter methylation levels of *IL20RB* were downregulated in tumor tissues and the degree of decline became more obvious with the increase of stage and grade (*p* < 0.001, Fig. 5B-E). Three gene expression profile datasets, GSE40435, GSE46699, and GSE53757, obtained from the GEO database were used to verify the differential expression of *IL20RB* in ccRCC. As shown in Fig. 5F-H, *IL20RB* expression was significantly higher in tumor tissues than in normal tissues. Moreover, we also analyzed the gene expression data and survival information of ccRCC patients downloaded from the ICGC database. The box plot and Kaplan-Meier curve again confirmed that *IL20RB* expression level was higher in tumor tissues and patients with overexpressed *IL20RB* had shorter overall survival (OS) (*p* = 0.013, Fig. 5I, J).

### In vitro validation of *IL20RB* in ccRCC

**Fig. 6:**
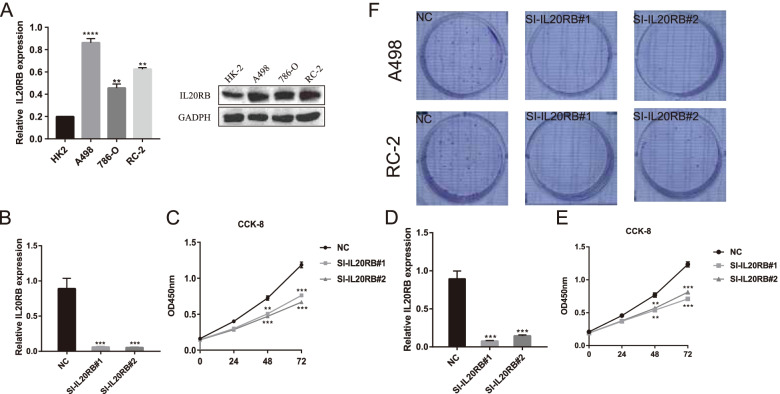
The overexpression of *IL20RB* in ccRCC cell lines and the proliferation of tumor cells after si-*IL20RB*. **A** QRT-PCR and western blot showed the overexpression of *IL20RB* in A498, 786-O, and RC-2 cell lines. **B, D** Detection of interference efficiency by qRT-PCR after knockdown of *IL20RB* in A498 and RC-2 cell lines, respectively. **C, E** The results of CCK-8 exhibited that knockdown of *IL20RB* significantly attenuated proliferation of A498 and RC-2 cells. **F** The quantities of A498 and RC-2 colony cells decreased significantly after si-*IL20RB.* *, *P* < 0.05; **, *P* < 0.01; ***, *P* < 0.001; **** *P* < 0.0001

To further validate the expression of *IL20RB*, different ccRCC cell lines, including A498, 786-O, and RC-2, and normal control HK2 cells were measured by qPCR and western blot. The result suggested that the mRNA and protein level of *IL20RB* were significantly higher in ccRCC cell lines, especially in A498 than in HK2 cells (*p* < 0.001, Fig. 6A). The experimental results were consistent with the conclusions of the bioinformatics analysis, indicating that *IL20RB* was highly expressed in ccRCC. Next, to explore the roles of *IL20RB* in ccRCC cell proliferation, si-*IL20RB* was transfected into A498 and RC-2 cells to downregulate the expression of *IL20RB*. Significant reduction of *IL20RB* expression was observed in Fig. 6B and D after si-*IL20RB* transfection (*p* < 0.001). Then, we detected cell proliferation levels using A498 and RC-2 cells with knockdown of *IL20RB*. Cell proliferation assays showed a remarkable decrease in proliferation levels after knockdown for 48 h and 72 h (Fig. 6C, E). Moreover, the results of the clone formation assay showed that the quantities of A498 and RC-2 cells were significantly lower in the si-*IL20RB* groups than that in the control groups (Fig. 6F). The above results indicated that knockdown of *IL20RB* significantly inhibited ccRCC cell proliferation.

### GSEA of different *IL20RB* expression levels

**Fig. 7:**
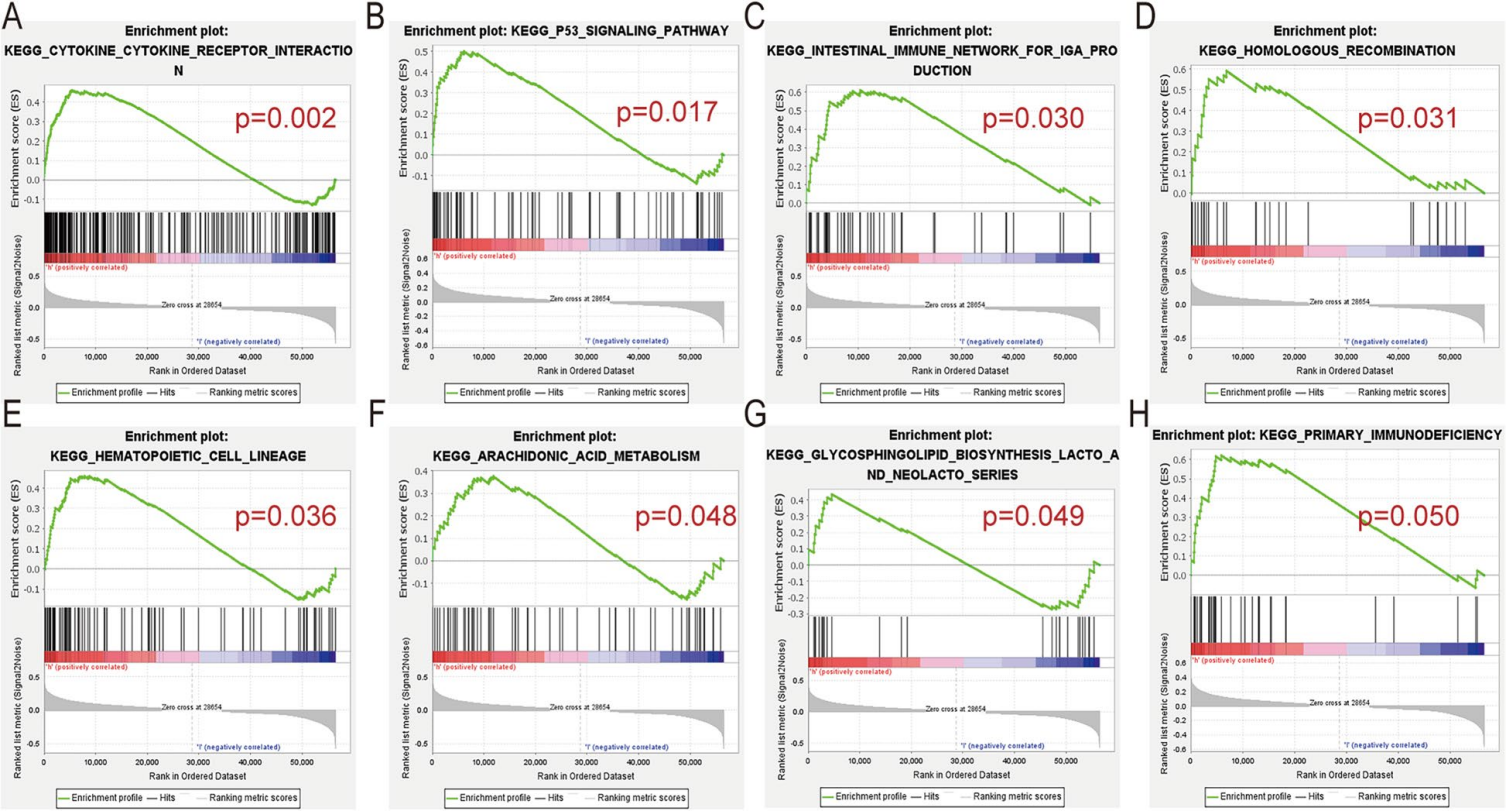
GSEA of *IL20RB* expression levels. **A** cytokine receptor interaction. **B** p53 signaling pathway. **C** immune network for IgA production. **D** homologous recombination. **E** hematopoietic cell lineage. **F** arachidonic acid metabolism. **G** glycosphingolipid biosynthesis of LACTO and NEOLACTO series. **H** primary immunodeficiency

To identify potential signaling pathways associated with *IL20RB* expression levels in ccRCC samples, GSEA of different *IL20RB* expression levels was undertaken. The results of GSEA were presented in Fig. 7A-H. High *IL20RB* expression levels were mainly enriched in cytokine-cytokine receptor interaction (CCRI), p53 signaling pathway, intestinal immune network (IIN) concerning IgA production, homologous recombination, hematopoietic cell lineage, arachidonic acid metabolism, primary immunodeficiency, glycosphingolipid biosynthesis of LACTO, and NEOLACTO series. We found that these signaling pathways that were enriched in the *IL20RB* overexpression groups were partly involved in the immune system.

### Association of *IL20RB* expression level with TIICs

**Fig. 8:**
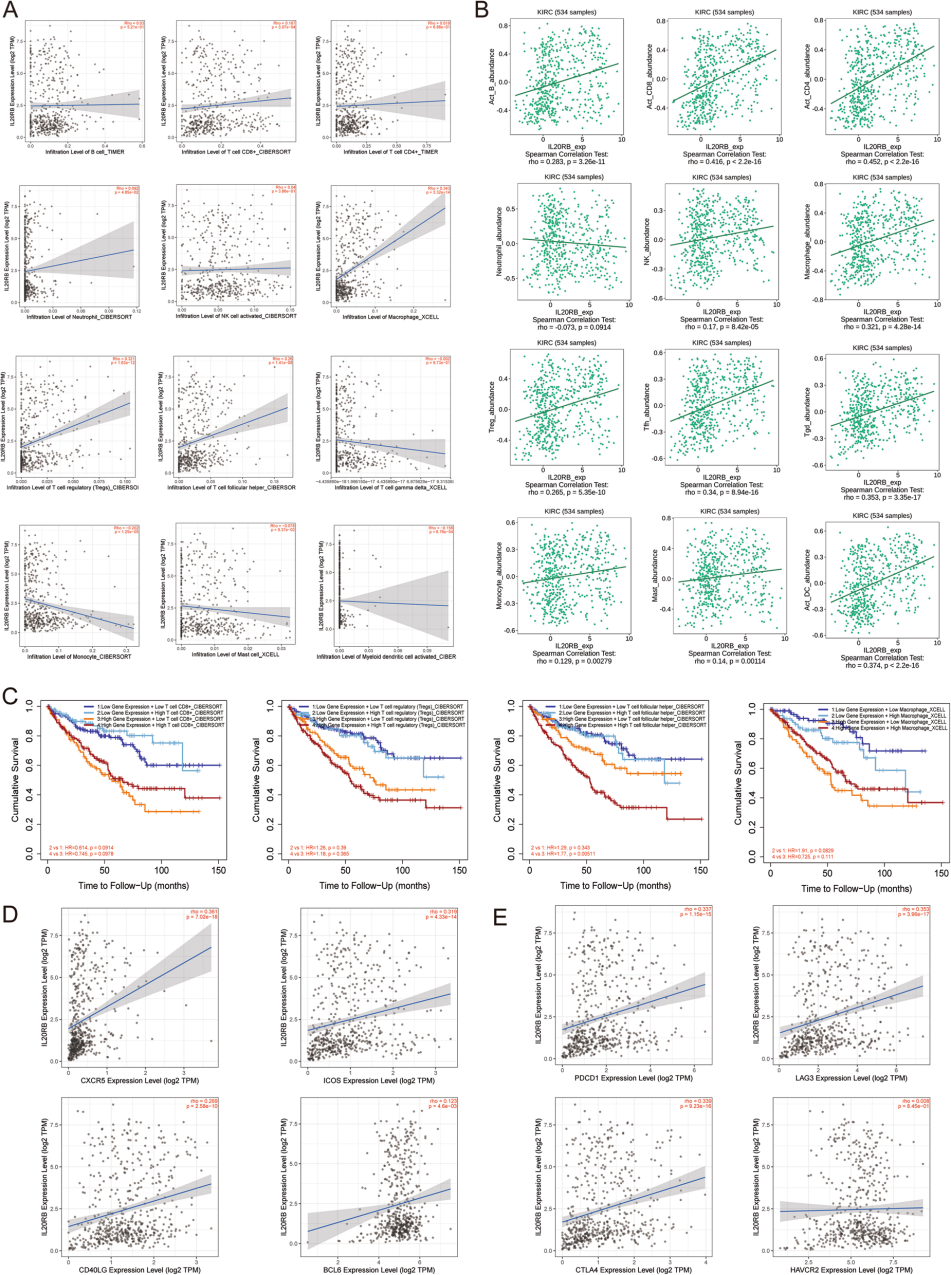
Relationship of *IL20RB* levels with TIICs and immune checkpoints. **A** The correlation analysis between *IL20RB* expression and infiltration levels of 12 TIICs via TIMER database. **B** Validation for the correlation between *IL20RB* expression and TIICs via TISIDB database. **C** Kaplan-Meier curves exhibited infiltration levels of Tfh cells, but not CD8+ T cells, Tregs and macrophages, were correlated with poor outcome in ccRCC. **D** Scatter plots of the associations between *IL20RB* and the markers of Tfh cells (CXCR5, ICOS, CD40LG and Bcl-6). **E** Scatter plots of the associations between *IL20RB* and immune checkpoints (PDCD-1, CTLA4, LAG3, and HAVCR2)

To investigate whether *IL20RB* expression levels were correlated with TIICs, the TIMER database was utilized to evaluate the association between *IL20RB* expression level and the abundance of 12 TIICs. The results showed that TIICs, including CD8+ T cells (*r* = 0.167, *p* = 3.07e− 04), regulatory T cells (Tregs) (*r* = 0.321, *p* = 1.63e− 12), T follicular helper (Tfh) cells (*r* = 0.26, *p* = 1.41e− 08), macrophages (*r* = 0.343, *p* = 3.32e− 14), monocytes (*r* = − 0.202, *p* = 1.25e− 05) and activated dendritic cells (*r* = − 0.158, *p* = 6.76e− 04) were significantly correlated with the *IL20RB* expression (Fig. 8A). To further confirm the results, we also analyzed the correlation between *IL20RB* expression and 12 TIICs in TISIDB database. As shown in Fig. 8B, *IL20RB* expression was correlated with active B cell (*r* = 0.283, *p* = 3.26e− 11), CD8+ T cells (*r* = 0.416, *p* < 2.2e− 16), CD4+ T cells (*r* = 0.425, *p* < 2.2e-16), Tregs (*r* = 0.235, *p* = 5.35e− 10), Tfh cells (*r* = 0.34, *p* = 8.94e− 16), T cell gamma delta (*r* = 0.353, *p* = 3.35e− 17), natural killer (NK) cells (*r* = 0.17, *p* = 8.42e− 05), macrophages (*r* = 0.321, *p* = 4.28e− 14), monocytes (*r* = 0.129, *p* = 0.00279), mast cells (*r* = 0.14, *p* = 0.00114) and activated dendritic cells (*r* = 0.374, *p* < 2.2e− 16). Finally, we found that the *IL20RB* expression had significantly positive correlation with the infiltration levels of CD8+ T cells, Tregs, Tfh cells and macrophages in both TIMER and TISIDB database. Then, we investigated whether there were statistical relationships between specific TIICs (CD8+ T cells, Tregs, Tfh cells and macrophages) and overall survival of ccRCC patients by TIMER database. Figure 8C showed that high infiltration level of Tfh cells was associated with poor outcome in ccRCC (*p* = 0.005). Additionally, we also analyzed the correlation between *IL20RB* and biomarkers of Tfh cells (CXCR5, ICOS, CD40LG and Bcl-6) as well as immune checkpoints (PDCD-1, CTLA-4, LAG3 and HAVCR2). The results showed that, except for HAVCR2, *IL20RB* expression had significantly positive correlations with these biomarkers (*p* < 0.001, Fig. 8D, E). Taken together, the *IL20RB* expression level was significantly associated with immune cell infiltration and immune biomarkers in ccRCC, which may have significant clinical implications.

## Discussion

Treatment of advanced ccRCC is mainly a challenge owing to the lack of effective treatment, and the 5-year survival rate of patients with advanced ccRCC is only 11.7% [1]. Immunotherapy, as a promising treatment, improves the survival rate of advanced ccRCC [21]. Immune checkpoint inhibitors, including pembrolizumab, nivolumab, and avelumab, have been used as first-line treatment modalities for advanced ccRCC, significantly improving the outcomes of patients with advanced ccRCC [9, 22, 23]. However, immunotherapy is only effective for a subset of patients. Thus, it is vital to identify further significant biomarkers to predict therapeutic efficacy before undergoing immunotherapy [24]. The TMB value is a promising predictor of the response of cancer patients after immunotherapy and may be used to determine treatment failure to immune checkpoint inhibitors in diverse types of cancer (e.g., melanoma, breast cancer, and small-cell lung cancer) [25–28]. However, whether it is associated with immunotherapy in ccRCC remains elusive, which motivates us to investigate the possible relationship of TMB value with the prognosis of cases with ccRCC. The results demonstrated that a high level of TMB was associated with higher tumor grades, advanced pathological stages, and worse survival outcomes, which was consistent with previously reported findings [29].

In the present study, *IL20RB* was identified as an independent prognostic factor for ccRCC; in addition, the IL20 subfamily, including IL19, IL20, and IL24, is involved in both amplified inflammatory responses and anti-inflammatory responses, such as tissue protection and regeneration [30–33]. IL19 can directly influence immune cells, IL20 has a significant effect on skin inflammation, and IL24 can promote apoptosis of different types of cancer [34–36]. *IL20RB*, as a subunit of the IL20 subfamily receptor, is involved in the inflammatory response and malignancies. To date, the function of *IL20RB* in ccRCC has not been explored. Hence, in our study, we verified that *IL20RB* was overexpressed in both ccRCC tissues and cells, and the ability of proliferation of ccRCC cells was inhibited after knockdown of the expression of *IL20RB.* Moreover, we conducted GSEA to identify possible pathways associated with *IL20RB* in ccRCC, and the results revealed that *IL20RB* might be involved in CCRI and IIN concerning IgA production and the p53 signaling pathway. The p53 gene is one of the most frequently mutated genes in human cancer, and the p53 signaling pathway is involved in many biological functions, e.g., reproduction, metabolism, cell cycle regulation, suppression of tumor expression, etc. [37–39]. It has been demonstrated that IIN concerning IgA production is involved in some types of cancer, such as hepatocellular carcinoma, which can be activated by CCR9, CCR10, and CXCR4 to promote tumor growth and metastases [40]. A previous study demonstrated that CCRI was a significant pathway of CXC chemokines in RCC, mediating the migration and localization of immune cells and influencing the prognosis of RCC patients [41].

Growing evidence has highlighted that immune cell infiltration is closely related to the prognosis of RCC cases [42]. In the present study, we found that CD8+ T cells, Tregs, Tfh cells, and Macrophages were overexpressed in patients with a high expression level of *IL20RB*. CD8+ T cells, Tregs and Macrophages have been proven to play an essential role in cancer development and metastasis [43–45]. However, our knowledge of the clinical implications of Tfh cells in cancer is still limited. Here, we found *IL20RB* expression level was correlated positively with the markers of Tfh cells, and overexpressed Tfh cells were correlated with poor prognosis of patients with ccRCC. Moreover, we also investigated the correlation between *IL20RB* and genes involved in immunotherapy, including PDCD-1, CTLA4, LAG3 and HAVCR2.The result showed that *IL20RB* expression was associated significantly with these immune checkpoints, suggesting that *IL20RB* was a potential therapeutic target correlated with tumor immunology.

In summary, our study explored the intrinsic correlation of the TMB value with clinicopathological parameters of ccRCC patients and elucidated that *IL20RB* was correlated with poor prognosis in ccRCC and could enhance the ability of proliferation of ccRCC cells. Moreover, the level of *IL20RB* was significantly related to immune cell infiltration and immune checkpoints in ccRCC, which may provide a new perspective for immunotherapy.

## Conclusions

In conclusion, the present study demonstrated that *IL20RB* was overexpressed in both ccRCC tissues and cells. Overexpression of *IL20RB* could enhance the viability of ccRCC cells and predict the poor prognosis of patients with ccRCC. Furthermore, the correlations between *IL20RB* and immune cell infiltration and immune checkpoints indicated a potential role for *IL20RB* in the immunotherapy of ccRCC.

## Supplementary Information

**Additional file 1.**

**Additional file 2.**

**Additional file 3.**

## Data Availability

The datasets used and/or analysed during the current study are available from the corresponding author on reasonable request.

## Abbreviations

*IL20RB*: Interleukin 20 receptor subunit beta
TMB: Tumor mutation burden
ICI: Immune cell infiltration
ccRCC: Clear cell renal cell carcinoma
GSEA: Gene set enrichment analysis
TIICs: qTumor-infiltrating immune cells
Tfh: T follicular helper
GO: Gene Ontology
OD: Optical density
TCGA: The Cancer Genome Atlas database
GEO: Gene Expression Omnibus
ICGC: International Cancer Genome Consortium
GEPIA: Gene Expression Profiling Interactive Analysis
TIMER: Tumor Immune Estimation Resource
TISIDB: Immune System Interaction Database
UALCAN: The University of ALabama at Birmingham CANcer data analysis Portal
