# Supplementary material for: Interleukin 20 receptor subunit beta (IL20RB) predicts poor prognosis and regulates immune cell infiltration in clear cell renal cell carcinoma
Source: BMC Genom Data. 2022 Jul 26;23:58. doi: 10.1186/s12863-022-01076-4 (PMC9327257; doi:10.1186/s12863-022-01076-4)
Supplement: Supplementary file 1 — Additional file 1. [file 12863_2022_1076_MOESM1_ESM.pdf]

Figure S1

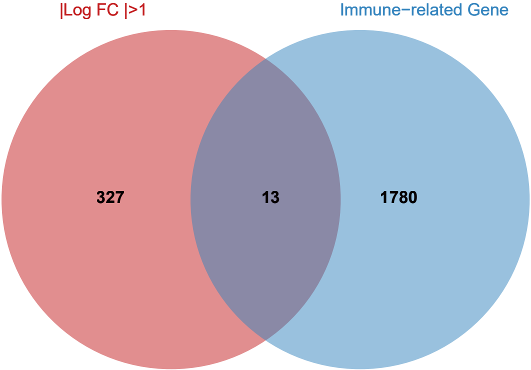

The Venn diagram showed 13 genes that were common between the DEGs and immune-related genes.
