# Supplementary material for: Interleukin 20 receptor subunit beta (IL20RB) predicts poor prognosis and regulates immune cell infiltration in clear cell renal cell carcinoma
Source: BMC Genom Data. 2022 Jul 26;23:58. doi: 10.1186/s12863-022-01076-4 (PMC9327257; doi:10.1186/s12863-022-01076-4)
Supplement: Supplementary file 2 — Additional file 2. [file 12863_2022_1076_MOESM2_ESM.pdf]

Figure S2

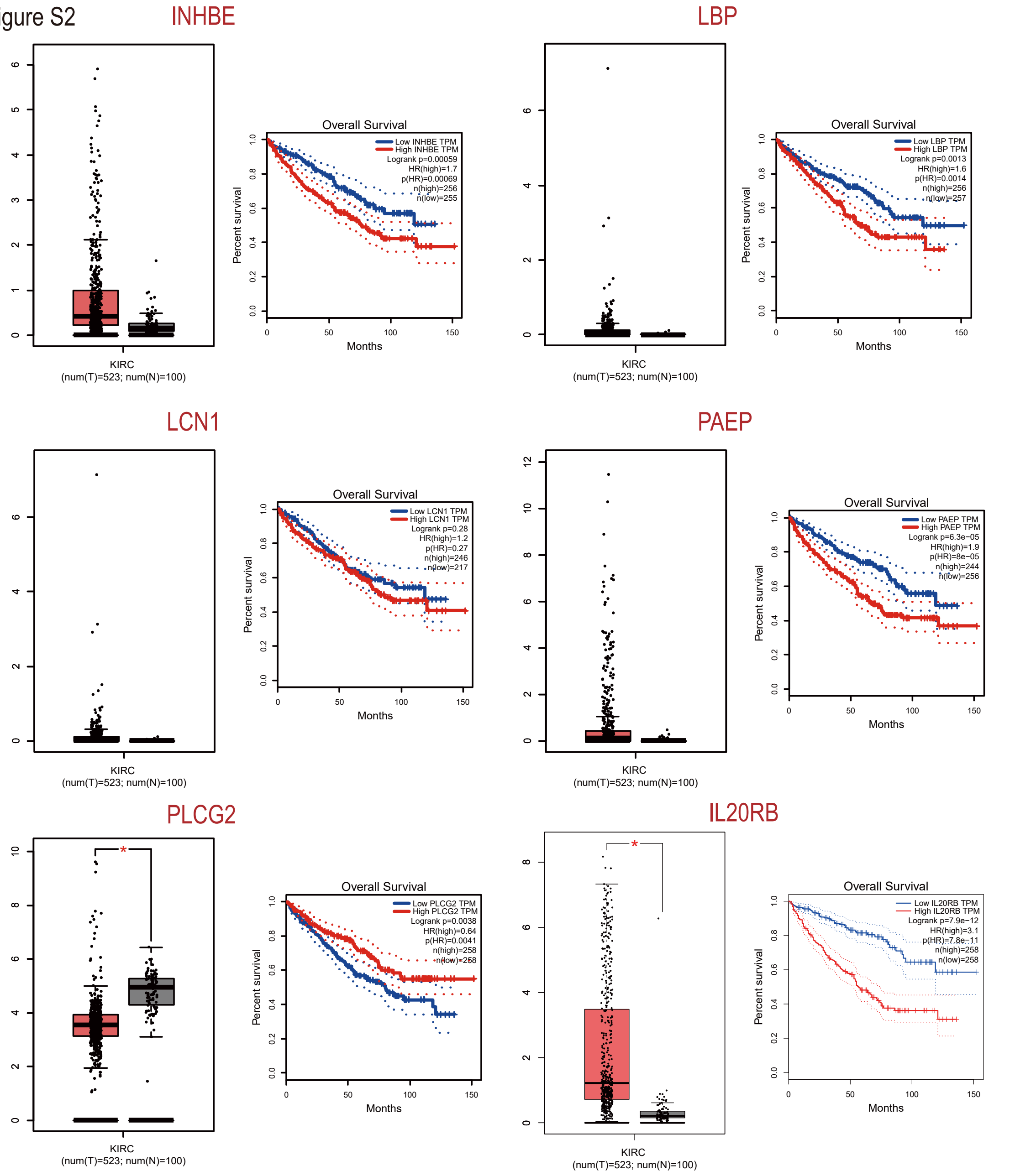

Expression levels and survival curves of INHBE, LBP, LCN1, PAEP, PLCG2 and IL20RB in ccRCC analyzed by GEPIA online database.
