# Supplementary figures and images for: Interleukin 20 receptor subunit beta (IL20RB) predicts poor prognosis and regulates immune cell infiltration in clear cell renal cell carcinoma
Source: BMC Genom Data. 2022 Jul 26;23:58. doi: 10.1186/s12863-022-01076-4 (PMC9327257; doi:10.1186/s12863-022-01076-4)

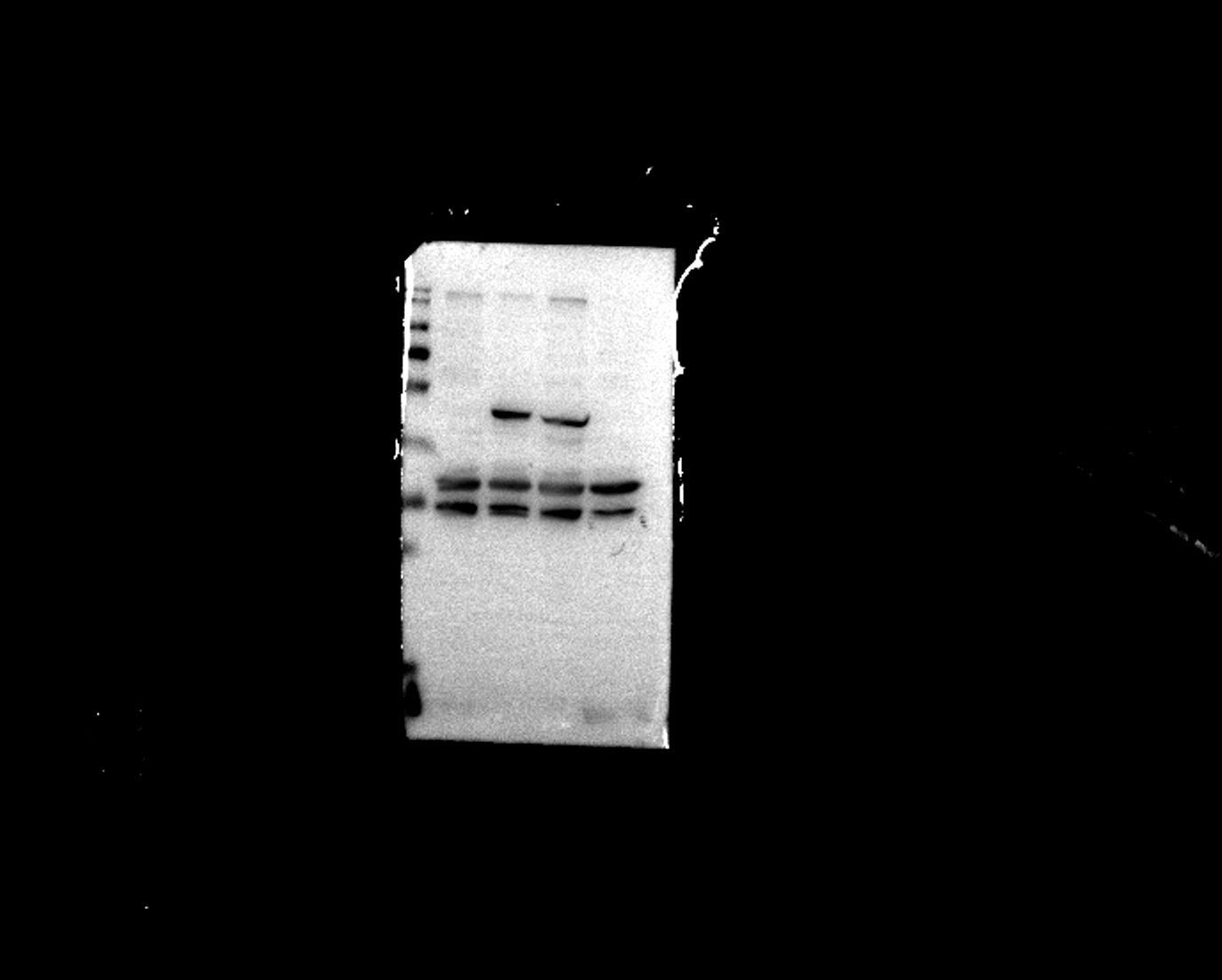

Supplement: Supplementary file 3 — Additional file 3. [file 12863_2022_1076_MOESM3_ESM.tif]
